# Multimodal Dynamics in Ionic Liquids Revealed by Molecular-Dynamics-Guided Multinuclear NMR Relaxation Analysis

**DOI:** 10.1021/acs.jpclett.6c02150

**Published:** 2026-07-13

**Authors:** Yanan Li, Florin Teleanu, Federico Civaia, Christoph Scheurer, Alexej Jerschow

**Affiliations:** † Department of Chemistry, 5894 New York University, New York, New York 10003, United States; ‡ ELI-NP, “Horia Hulubei” National Institute for Physics and Nuclear Engineering, 30 Reactorului Street, Bucharest-Magurele 077125, Ilfov, Romania; § Fritz Haber Institute of the Max Planck Society, Berlin 14195, Germany

## Abstract

Ionic liquids are complex liquids characterized by high viscosity and high polarity. Their dynamics are of interest due to their use in many fields that require unconventional solvent environments. Here, we develop an MD-based forward-prediction framework to calculate NMR relaxation rates in the ionic liquid 1-butyl-3-methylimidazolium tetrafluoroborate, [bmim]­[BF_4_], and investigate their temperature and magnetic-field dependence. We show that the combination of intra- and intermolecular dipolar couplings and chemical shift anisotropy interactions allows accounting for the experimentally observed nuclear spin lifetimes when considering multimodal relaxation processes. A regularized inverse Laplace transformation of MD-derived interactions reveals generally bimodal motional processes active in both inter- and intramolecular effects. The presence of two main motional processes may be somewhat surprising for intramolecular effects, especially in the symmetric [BF_4_]^−^ unit, but can be attributed to the restricted motion due to the local environment. We also highlight that the Bloembergen–Purcell–Pound (BPP) approach that is often used to characterize average motional correlation times is not reliable in most situations encountered with realistic systems. Consequently, we argue that achieving quantitative agreement between predicted and measured NMR relaxation rates offers a more robust route to extracting structural and dynamical information from complex liquids.

## Graphical abstract

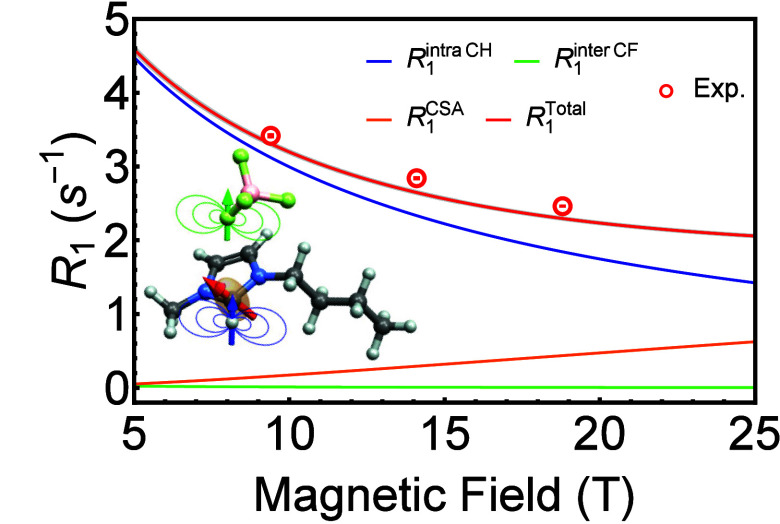

Ionic liquids (ILs) are widely known as “designer solvents” due to the flexible combination of organic cations with inorganic or organic anions, typically exhibiting melting points below that of water,^1,2^ negligible vapor pressure, high thermal stability, and intrinsic ionic conductivity which renders them attractive alternatives to conventional solvents and promising materials for electrochemical applications.^3^ The dynamic properties of ILs have therefore attracted sustained attention, as they play a central role in determining task-specific performance. In particular, translational and rotational molecular motions govern key processes such as ion transport, viscosity, and electrochemical response.^4–8^ Multinuclear NMR relaxation techniques are particularly suited to derive site-specific information on reorientational dynamics, enabling a detailed atomic picture of molecular motion.^9–11^ However, extracting accurate time scales of molecular dynamics from such experiments is not trivial and a naive application of classical NMR theory can lead to inaccurate results. To gain further insight, experimentally derived dynamic time scales can be linked with values predicted from Molecular Dynamics (MD) simulations,^12–14^ but, as we will show below, NMR relaxometry can only unravel dynamics on specific time scales imposed by the field and the magnetic response of the targeted nucleus.

The Bloembergen–Purcell–Pound (BPP) theory is widely used to extract correlation times from nuclear spin relaxation experiments, assuming molecules undergo unrestricted isotropic tumbling in solution.^15–17^ This assumption implies, for example, that the autocorrelation function of the intramolecular dipole–dipole (DD) interaction in an ^1^H–^13^C pair has a simple, monoexponential form *G*_R_^BPP^(*t*) = $\frac{1}{5}A_{0}\exp\left(-t/\tau_{R,C}\right)$ , where *A*_0_ is proportional to the dipole coupling strength and τ_R,C_ is known as the rotational correlation time. By performing the Fourier Transform (FT) of *G*_R_^BPP^(*t*), the classical Lorenzian-shaped spectral density *J*_R_^BPP^(ω) characterizes the efficacy of the DD interaction to induce relaxation at a certain frequency ω according to i.e.,

1 $$J_{BPP}\left(\omega \right)=\frac{2}{5}A_{0}\frac{\tau_{R,C}}{1+\omega^{2}\tau_{R,C}^{2}}$$

which one uses to build the autorelaxation rate of individual spins as $R_{1,\mathrm{BPP}}^{H/C}=J_{\mathrm{BPP}}\left(\omega_{H}-\omega_{C}\right)+3J_{\mathrm{BPP}}\left(\omega_{H/C}\right)+6J_{\mathrm{BPP}}\left(\omega_{H}+\omega_{C}\right)$ . Assuming this is the sole source of relaxation, one can then derive dynamic parameters by running temperature-dependent experiments of the spin–lattice relaxation time *T*_1_. A local minimum of *T*_1_ value satisfies the condition ω_0_τ_R,C_ ≈ constant, with characteristic ω_0_τ_R,C_ values of ∼0.923 for ^1^H and ∼0.791 for ^13^C.^11,18,19^ Once the correlation time at this characteristic point is determined, the prefactor in the BPP equation can be evaluated, allowing the correlation time τ_c_ to be estimated over the entire temperature range.^11^ Alternatively, field-cycling experiments can map spectral density components of different relaxation mechanisms by measuring polarization lifetimes at multiple fields and extract dynamic parameters by fitting adequate theoretical models.^20–23^ Both these methods rely on the BPP model which has some significant drawbacks:

(1) Locating the *T*_1_ minimum often requires measurements at sufficiently low temperatures, which may be experimentally inaccessible due to instrumental/sample constraints.
(2) Interpreting field-dependent relaxation rates as a cumulative effect of multiple relaxation contributions often leads to a large number of fitting variables making the method prone to overfitting.
(3) The BPP model assumes DD interactions to be the dominant relaxation mechanism and therefore does not allow for a quantitative or qualitative separation of other relaxation contributions such as chemical shift anisotropy (CSA) or quadrupolar interaction.
(4) Most importantly, molecules in isotropic conditions are often characterized by broadly distributed dynamics,^24^ especially ILs,^25,26^ which violate the monoexponential assumption underlying the BPP model.

Thus, the following question arises: *What is the physical interpretation of a correlation time derived experimentally from the scaling* $\tau_{R,C}\approx \frac{\mathrm{constant}}{\omega_{0}}$ *and how accurate is this approach?* Because this method effectively assumes a single-Lorentzian spectral density it may therefore reflect model artifacts rather than genuine molecular dynamics. As demonstrated by Asthagiri et al.,^27^ reduced molecular symmetry and flexibility give rise to heterogeneous dynamics, resulting in a broad, multiexponential decay of the autocorrelation functions, even for individual pairs of dipolar coupling interactions in water. Therefore, the autocorrelation function *G*(*t*) is more adequately expressed as a superposition of exponential decays,^27^

2 $$G\left(t\right)=\frac{1}{5}A_{0}\int_{0}^{\infty }g\left(\tau \right)\;\exp\left(-t/\tau \right)\;d\tau$$

where *g*(τ) represents the normalized distribution of correlation times τ. The extraction of *g*(τ) from the autocorrelation function constitutes an inverse Laplace transform problem, which is well-known to be ill-conditioned and highly sensitive to noise (eq 3),

3 s=Kg+e

where **s**, **K**, **g**, and **e** are the vectorized forms of the autocorrelation function, the kernel, the distribution function, and the noise, respectively. Tikhonov regularization is employed to stabilize the inversion. By solving eq 4 under suitable convergence criteria,^28^ the recovered distribution *g*(τ) approaches the physically meaningful solution that best represents the underlying dynamics,

4 $$ĝ=\arg\min_{g}\left\{∥Kg-s{∥}_{2}^{2}+\alpha_{00}^{2}∥g{∥}_{\Lambda_{2}}^{2}\right\}$$

where α_00_ is the regularization parameter which controls the regularization strength.

The regularized inverse Laplace transform (RILT) approach offers distinct advantages for the analysis of complex relaxation dynamics of nuclear spins. It does not rely on a priori assumptions such as monoexponential behavior,^29^ a predefined number of dynamical components in multiexponential models,^30,31^ or phenomenological stretched-exponential forms with empirically chosen stretching factors.^32^ Instead, it enables a data-driven reconstruction of a continuous distribution of correlation times, thereby providing a more flexible description of heterogeneous dynamics. Recently, Padé–Laplace inversion has also been explored as an alternative approach for resolving complex relaxation.^33,34^ Here, we employ the RILT framework for its robustness toward noisy simulation-derived autocorrelation functions. Correspondingly, the spectral density function can be determined from the Fourier transform of the autocorrelation function (eq 2) and expressed as^27^

5 $$J\left(\omega \right)=\frac{2}{5}A_{0}\int_{0}^{\infty }g\left(\tau \right)\frac{\tau }{1+{\left(\omega \tau \right)}^{2}}\;d\tau$$

accounting for the cumulative effects of different dynamical regimes. As we describe below, this approach grants better predictions of relaxation rates than describing the spectral density as consisting of only a single Lorentzian function $J_{\mathrm{BPP}}\left(\omega \right)=\frac{2}{5}\frac{\tau_{C}^{\mathrm{av}}}{1+{\left(\omega \tau_{C}^{\mathrm{av}}\right)}^{2}}$ with an averaged correlation time commonly derived as $\tau_{C}^{\mathrm{av}}=\int_{0}^{\infty }G_{R}^{\mathrm{BPP}}\left(t\right)/G_{R}^{\mathrm{BPP}}\left(0\right)\;dt$ which we show cannot be straightforwardly linked to the experimentally derived value using ω_0_τ_C_ ≈ constant.

As a case study, we investigate the ^13^C and ^19^F nuclear polarization lifetimes in 1-butyl-3-methylimidazolium tetrafluoroborate ([bmim]­[BF_4_]) where the two ions are characterized by very different rotational and translational dynamics due to their distinct shape and molecular weight. The ^13^C and ^19^F 1D NMR spectra of [bmim]­[BF_4_] are shown in Figure 1(a) and (d), respectively. To achieve high resolution and optimal signal-to-noise ratios, ^13^C resonances were recorded using a power-gated decoupling sequence, which provides efficient suppression of ^1^H–^13^C J-coupling interactions. As a result, eight distinct carbon signals are observed, corresponding to the eight chemically inequivalent carbon sites of the [bmim] molecule. In the ^19^F spectrum (no decoupling), two manifolds are detected arising from the secondary isotope effect^35^ of the two boron isotopes ^10/11^B with a natural abundance ratio of *N*_10B_: *N*_11B_ ≈ 1:4 and nuclear spin values of *S*_10B_ = 3 and *S*_11B_ = 3/2, respectively.

**Figure 1:**
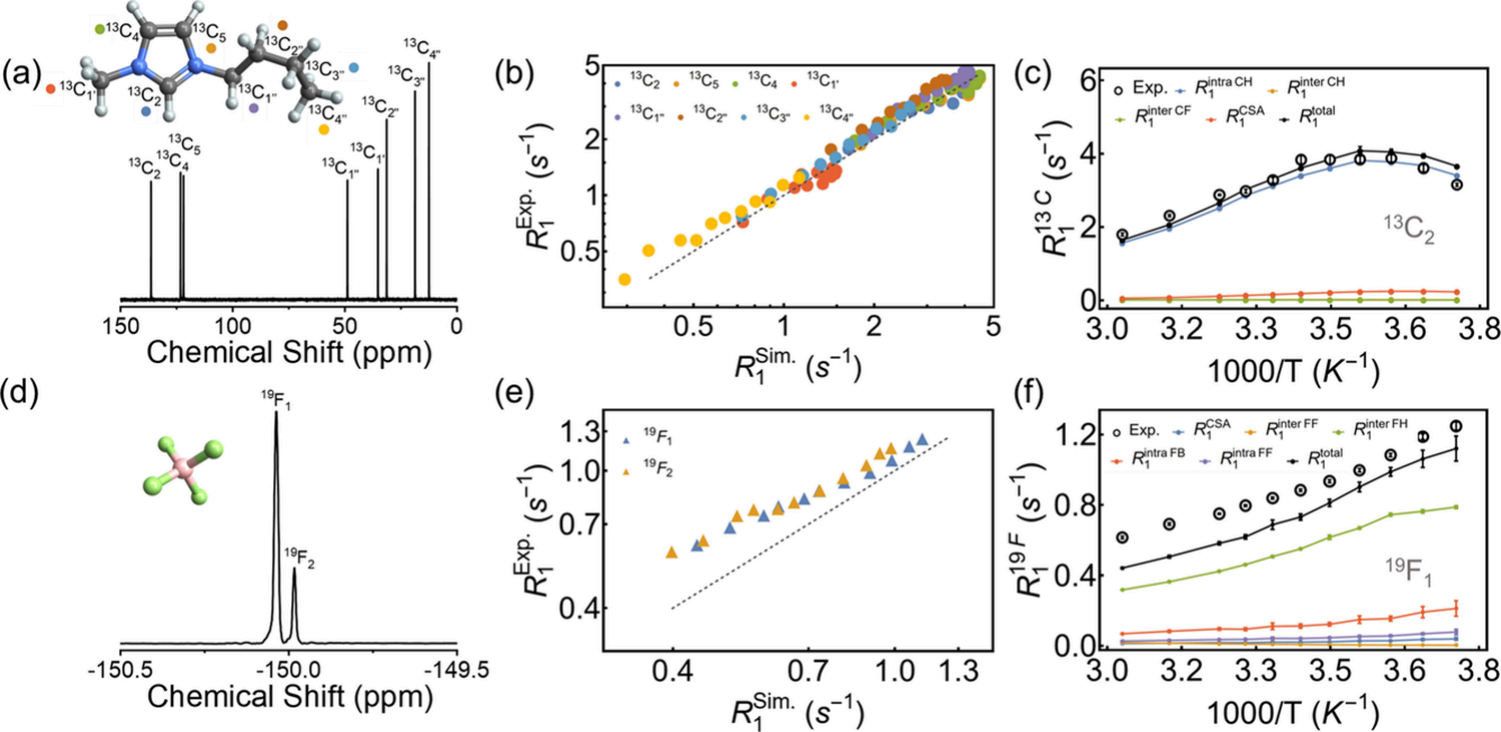
(a) 1D ^13^C­{^1^H} NMR spectrum (^13^C spectrum with ^1^H decoupling) of [bmim]­[BF_4_] with peak assignments (inset: molecular structure and atom labeling). (b) Cross-plot (in log-scale) of experimental and simulated ^13^C spin–lattice relaxation rates at 9.4 T at multiple temperatures ranging between 270–330 K. Color coding corresponds to the carbon-site labeling shown in the inset of panel (a). (c) Temperature dependence of the identified relaxation contributions for the ^13^C_2_ site. The sum of individual contributions matches the total relaxation rate for ^13^C in [bmim]­[BF_4_]. (d) 1D ^19^F NMR spectrum of [bmim]­[BF_4_] with peak assignments. The ^19^ *F*_1_ peak corresponds to the ^19^F nuclei coupled to ^11^B, whereas ^19^ *F*_2_ corresponds to ^19^F nuclei coupled to ^10^B. (e) Cross-plot (in log-scale) of experimental and simulated ^19^F spin–lattice relaxation rates at 9.4 T at multiple temperatures ranging between 270–330 K. (f) Temperature dependence of the identified relaxation contributions for the fluorine ^19^ *F*_1_ site. The sum of individual contributions reproduces the temperature trend of the total relaxation rate of ^19^F in [BF_4_]^−^ to within a relatively constant offset value.

The overall ^13^C and ^19^F spin–lattice relaxation rates arise from the cumulative contributions from different mechanisms such as DD interactions and CSA contribution. The DD contributions arise from both intramolecular and intermolecular interactions, mostly due to nuclei featuring large gyromagnetic constants such as ^1^H and ^19^F. In order to fit experimental data, ideally, one would need to consider all possible relaxation contributions described by specific analytical models, each with at least two independent fitting variables: the strength of the relaxation interaction (coupling constant) and the time scale on which that interaction is being modulated (correlation time). For highly concentrated systems composed of complex molecules bearing multiple nuclear species such as ionic liquids, this approach is unfeasible as the number of relaxation interactions is large and the classical analytical models used for fitting rely on the assumptions that nuclei tumble as hard spheres. Given the anisotropic molecular shape and strong Coulomb interactions between ions in such systems, standard NMR models describing relaxation interactions modulated by rotational diffusion (BPP model) or translational diffusion (Hwang-Freed^36^ model) are not expected to capture the heterogeneous and complex dynamics specific to ionic liquids (see Figure S1). To gain deeper insight into the rotational and translational tumbling of [bmim]­[BF_4_] leading to relaxation, molecular dynamics (MD) simulations were performed, and the corresponding autocorrelation functions *G*(*t*) were evaluated to establish a direct connection between microscopic motions and experimentally observed NMR relaxation rates. The following contributions have been identified for the relaxation of ^13^C and ^19^F nuclear spins:

6 $$R_{1}^{13C,total}=R_{1}^{intra,CH}+R_{1}^{inter,CH}+R_{1}^{inter,CF}+R_{1}^{CSA}$$

7 $$R_{1}^{19F,total}=R_{1}^{intra,FF}+R_{1}^{intra,FB}+R_{1}^{inter,FF}+R_{1}^{inter,FH}+R_{1}^{CSA}$$

where $R_{1}^{intra,IS}$ and $R_{1}^{inter,IS}$ correspond to the contribution of intra- and intermolecular DD interactions between spins I and S, respectively, while $R_{1}^{CSA}$ represents the contribution of the chemical shift anisotropy mechanism.

The autocorrelation function of the DD interaction experienced by a spin I due to coupling to a spin S is^17,27^ given by

8 $$G_{Dipolar}\left(t\right)=\frac{1}{3}{\left(\frac{\mu_{0}\hbar \gamma_{I}\gamma_{S}}{4\pi }\right)}^{2}S\left(S+1\right)\times {\left\langle \underset{P_{IS}}{\sum }\frac{P_{2}\left[{û}_{IS}\left(t_{0}\right)\cdot {û}_{IS}\left(t_{0}+t\right)\right]}{r_{IS}^{3}\left(t_{0}\right)r_{IS}^{3}\left(t_{0}+t\right)}\right\rangle }_{origins,residues}$$

where μ_0_ is the magnetic permeability, *ℏ* is the reduced Planck constant, γ_I,S_ are the gyromagnetic constants of the two spins, *P*_IS_ is the set of {I,S} spin pairs, *P*_2_ is the second Legendre polynomial, ${û}_{\mathrm{IS}}$ is the unit vector connecting spins I and S, and *r*_*IS*_ denotes the corresponding time-dependent internuclear distance derived from MD trajectories. The angular brackets indicate averaging over time origins and molecular residues. In the case of the dipole–dipole interaction, the prefactor *A*_0_ in eqs 2 and 5 is equal to *G*_Dipolar_(0). Depending on the nature of spin pairs, the contribution to the total relaxation rate of spin I due to the dipolar coupling to spin S with different gyromagnetic ratio is expressed as

9 $$R_{1}^{I}=J\left(\omega_{I}-\omega_{S}\right)+3J\left(\omega_{I}\right)+6J\left(\omega_{I}+\omega_{S}\right),\gamma_{I}\neq \gamma_{S}$$

for heteronuclear spin pairs, while in the homonuclear case, cross-relaxation effects need to be considered,^17,37^ leading to the following expression:

10 $$R_{1}^{I}=3\left(J\left(\omega_{I}\right)+4J\left(\omega_{I}+\omega_{S}\right)\right),\gamma_{I}=\gamma_{S}$$

For evaluating CSA relaxation contributions, we performed static DFT calculations on multiple MD snapshots, selecting only one residue at a time (see the Supporting Information for a more-detailed description of the workflow). The symmetric and antisymmetric tensor components contribute according to^38^

11 $$R_{1}^{sym}=\frac{1}{4}{\left(\omega_{0}\sqrt{\frac{3}{2}}∥\sigma_{sym}{∥}_{F}\right)}^{2}\int_{0}^{\infty }g\left(\tau \right)\frac{\tau }{1+{\left(\omega \tau \right)}^{2}}\;d\tau$$

12 $$R_{1}^{anti}=\frac{1}{6}{\left(\omega_{0}∥\sigma_{anti}{∥}_{F}\right)}^{2}\int_{0}^{\infty }g\left(\tau \right)\frac{3\tau }{1+{\left(3\omega \tau \right)}^{2}}\;d\tau$$

where σ_sym_ and σ_anti_ denote the traceless symmetric and antisymmetric components of the CSA tensor, respectively. The Frobenius norm ∥σ∥_F_ is defined as the square root of the sum of the squares of all tensor elements. The parameters 3τ and τ correspond to first- and second-rank correlation times.

Adding all these contributions, the proposed approach achieves excellent agreement with the experimental data (Figure 1(b), (e)). The analysis reveals that intramolecular DD interactions dominate the relaxation of ^13^C nuclei (Figure 1(c)). In contrast, intermolecular DD contributions are negligible, which can be attributed to the larger average distances between the carbon nuclei and surrounding intermolecular ^1^H and ^19^F spins, leading to substantially weaker dipolar couplings. Figure 1(f) indicates that intermolecular ^19^F–^1^H dipolar interactions constitute the dominant relaxation pathway of ^19^F nuclear spins, in contrast to previous results which considered only the intramolecular ^19^F–^19^F DD mechanism as the dominant relaxation interaction leading to overestimating rotational correlation times of the BF_4_ unit.^39^ The ^11^B–^19^F_1_ dipolar coupling is slightly stronger than the ^10^B–^19^F_2_ coupling, which accounts for the observed difference in relaxation rates for the two peaks (see Figure 1(e) and Figure S10).

Even though the relaxation of ^13^C is dominated by intramolecular DD interactions, particularly those involving directly bonded hydrogen atoms, the correlation time one can derive experimentally using ω_0_τ_C_ ≈ constant provides a limited perspective of the overall dynamics. As an instructive example, let us consider the relaxation behaviors of two carbon atoms whose dipolar interactions are modulated on different time scales. Interactions involving the ^13^C_2_ of the imidazolium ring are expected to be modulated on the slow time scale of [bmim] rotational diffusion, while dipolar interactions of the ^13^C_1′_ methyl group should be modulated faster due to rapid internal rotation around the C–N bond. Additionally, as shown in Figure 2(a), the ^13^C_1′_ site has three bonded ^13^C–^1^H pairs, whereas the ^13^C_2_ site has only one. Experimentally, the maxima of *R*_1_ values for both sites occur at nearly the same temperature (around 285 K), suggesting that the two carbons experience the same dynamical time scales for the intramolecular DD interaction (see Figure 2(f)). Under this assumption, one might expect that the relaxation rate of ^13^C_1′_ should be approximately three times larger than that of ^13^C_2_ due to higher proton density. However, the experimental results show the opposite trend, which appears counterintuitive. This discrepancy cannot be explained by the conventional BPP model, as evidenced by the significant deviation between the anticipated monoexponential decorrelation of the dipole–dipole interaction and the actual dynamics (Figure 2(b)), nor by intermolecular DD contributions which are predicted to be very similar for the two carbon sites (Figure 2(c)). Therefore, one *cannot* link the experimentally inferred correlation time $\tau_{C}^{exp}\approx 0.791/\omega_{0}\approx 1.25$ ns at 285 K and 9.4 T, corresponding to the maximum observed rate for both carbon sites (Figure 2(f)), with the naively derived value from MD simulations as $\tau_{C}^{av}=\int_{0}^{\infty }\frac{G\left(t\right)}{G\left(0\right)}\;dt$ resulting in $\tau_{C}^{C_{2}}\approx 771$ ps and $\tau_{C}^{C_{1^{\prime }}}\approx 133$ ps, respectively. As shown in the ILT plots of the corresponding ACFs (Figure 2(d)), the underlying dynamics actually consist of two components, one peaking at around 1 ps and the other at approximately 1 ns, with different relative contributions for the two carbon interactions. However, NMR relaxation measurements are sensitive only to those components that can induce spin transitions on time scales close to 1/ω_0_ (here, ω_0_/2π ≈ 100 MHz for ^13^C nuclei at B_0_ = 9.4 T), rendering fast dynamics on the picosecond scale unobservable (Figure 2(e)). By scaling the distribution of correlation times with the frequency-dependent Lorenzian factor (eq 5), the fast-tumbling components become negligible, despite having a significant contribution to the RILT plot of the ^13^C_1′_ nucleus. Thus, only the slowly modulated components can lead to significant relaxation and can be observed experimentally, providing an incomplete picture of the full dynamics. Although these slow components share similar characteristic time scales for the two carbon interactions (around 1 ns), their amplitudes differ substantially due to site-specific internal motions, which, in turn, modulates the relative amplitude of relaxation rates. Our model explicitly accounts for these multicomponent dynamical contributions and therefore provides a more accurate description of the relaxation behavior than the conventional BPP framework.

**Figure 2:**
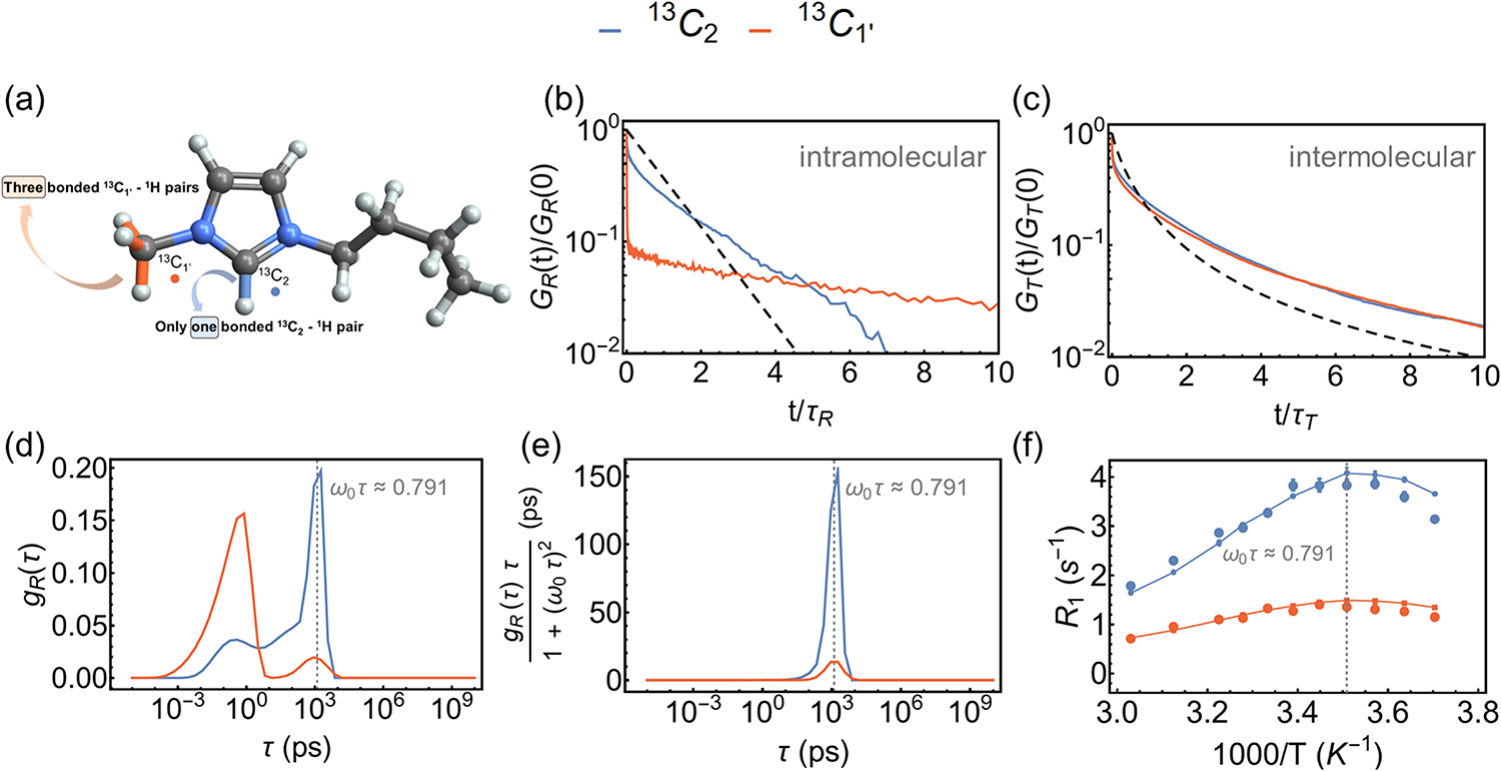
(a) Molecular structure of the [bmim]^+^ cation highlighting the ^13^C_2_ and ^13^C_1′_ sites and their bonded ^13^C–^1^H pairs used for the intramolecular dipole–dipole relaxation analysis. (b) Normalized autocorrelation functions of the intramolecular ^13^C–^1^H dipole–dipole interactions for the ^13^C_2_ and ^13^C_1′_ sites at 285 K, dashed line represents a fit based on the BPP model. (c) Normalized autocorrelation functions of the intermolecular ^13^C–^1^H dipole–dipole interactions for the ^13^C_2_ and ^13^C_1′_ sites at 285 K, dashed line corresponds to a fit based on the Hwang–Freed model.^36^ (d) RILT spectra of the intramolecular dipole–dipole interactions for the ^13^C_2_ and ^13^C_1′_ sites. (e) Spectral density distributions derived from the RILT spectra for the ^13^C_2_ and ^13^C_1′_ sites. (f) Temperature dependence of the experimental (big circles) and RILT-based MD-derived (full line) spin–lattice relaxation rates for the ^13^C_2_ and ^13^C_1′_ in [bmim]­[BF_4_]. The theoretical maximum relaxation rate (small circles) value is achieved at 285 K.

Figure 3 further examines ^13^C relaxation components measured at different magnetic fields. The consistent reproduction of the experimental data across three fields confirms the robustness of the RILT-based analysis and enables a quantitative separation of DD and CSA contributions. The analysis reveals the different field-scaling of DD and CSA contributions: the former decreases at higher fields as it approaches the “slow tumbling regime” satisfying ω_0_τ_C_ ≫ 1, while the latter increases as the CSA coupling strength is proportional to $B_{0}^{2}$ .^17^ The three carbon atoms on the imidazolium ring exhibit the strongest CSA contributions among all carbon sites, while the two aliphatic carbon atoms bonded to the nitrogen atoms display only minor CSA contributions. The carbon atoms located in the terminal region of the *n*-butyl chain, far from the imidazolium ring, are predominantly governed by intramolecular ^13^C–^1^H DD interactions, with negligible CSA contributions at all fields. Taken together, these results establish a clear hierarchy of relaxation mechanisms: intramolecular ^13^C–^1^H DD interactions dominate the overall relaxation, whereas CSA effects become increasingly significant for highly deshielded carbons within the imidazolium ring at higher magnetic fields. Consequently, at higher fields, the equality ω_0_τ_R,C_ ≈ 0.791 cannot be used to derive accurate tumbling rates at the maximum ^13^C relaxation rate value measured at a specific temperature, as the main intramolecular ^13^C–^1^H DD mechanism is supplemented by the field-dependent CSA contribution.^19^

**Figure 3:**
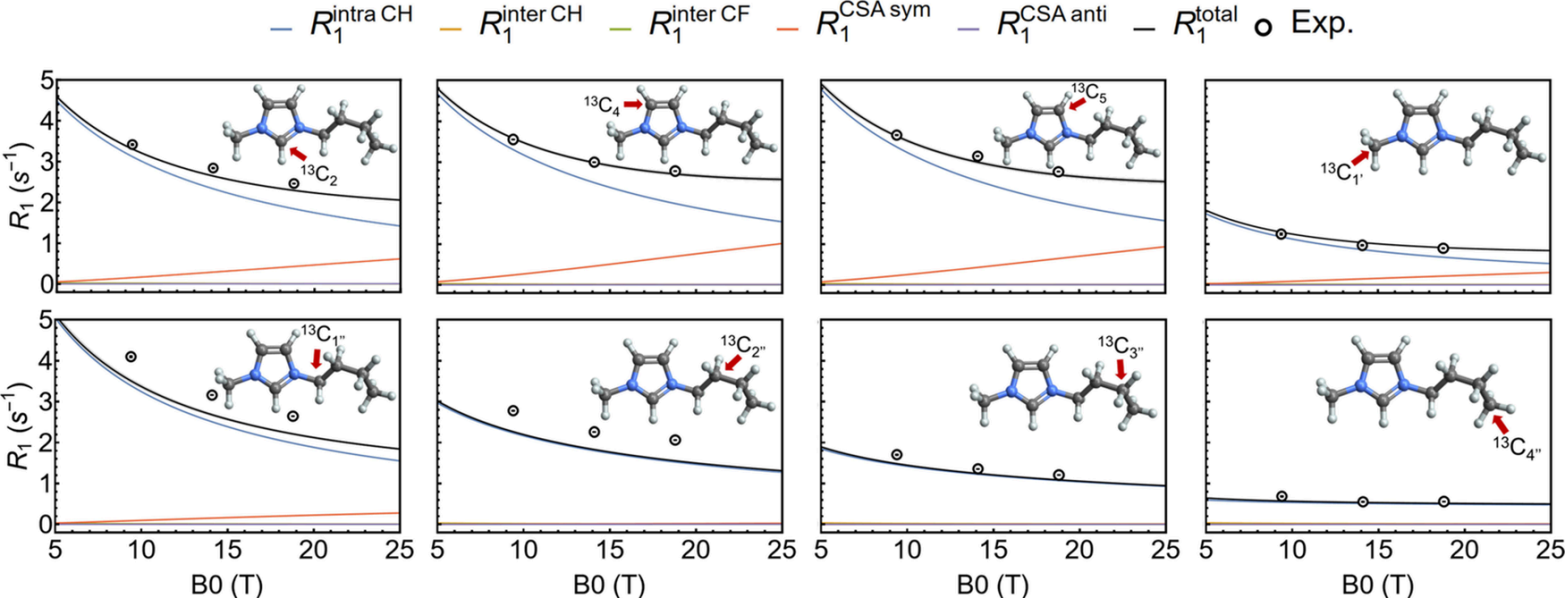
Field dependence of the RILT-based MD-derived relaxation contributions (lines) whose sum matches well the measured ^13^C relaxation rates (circles) in [bmim]­[BF_4_] at fields of at 9.4, 14.1, and 18.8 T and 300 K.

More importantly, although NMR relaxation measurements are intrinsically insensitive to dynamical processes far from the characteristic time scale τ ≈ 1/ω_0_, the combination of NMR experiments with MD simulations allows for a more comprehensive perspective of the broad dynamical landscape of the [bmim]­[BF_4_] system. In particular, the RILT analysis of the MD-derived correlation functions characteristic to intra- and intermolecular DD interactions resolves multiple peaks, enabling the underlying dynamical processes in the ILs to be clearly distinguished. As shown in Figure 4, the tumbling motion of both the cation and the anion exhibits multiple dynamical components. Notably, even the highly symmetric [BF_4_]^−^ anion displays two-component rotational dynamics, highlighting the limitations of the single-correlation-time assumption in the BPP model. A similar multiplicity of time scales is also observed for the relative motion between the ions. This behavior is consistent with the two or three relaxation modes reported by Nakamura et al. in dielectric relaxation studies,^40^ as well as with the observations of Endo and co-workers.^41^ These results suggest that the rotational dynamics of the [bmim]^+^ cation and [BF_4_]^−^ anion arise from a combination of a slow overall tumbling motion, corresponding to reorientation of the molecular frame, and faster internal motions associated with reorientation of the spin–spin vectors about the molecular frame.^42,43^ As demonstrated by Woessner,^42,43^ internal motions can partially average the dipolar interaction, thereby reducing the relaxation rate expected from overall tumbling alone. This interpretation is consistent with our observations. In the present system, the internal motions occur on time scales that lie largely outside the NMR relaxation observable window at this particular field and therefore do not contribute to the measured relaxation rate. This explains why certain multimodal motions detected by dielectric spectroscopy are not fully captured by NMR relaxation measurements alone.^40^

**Figure 4:**
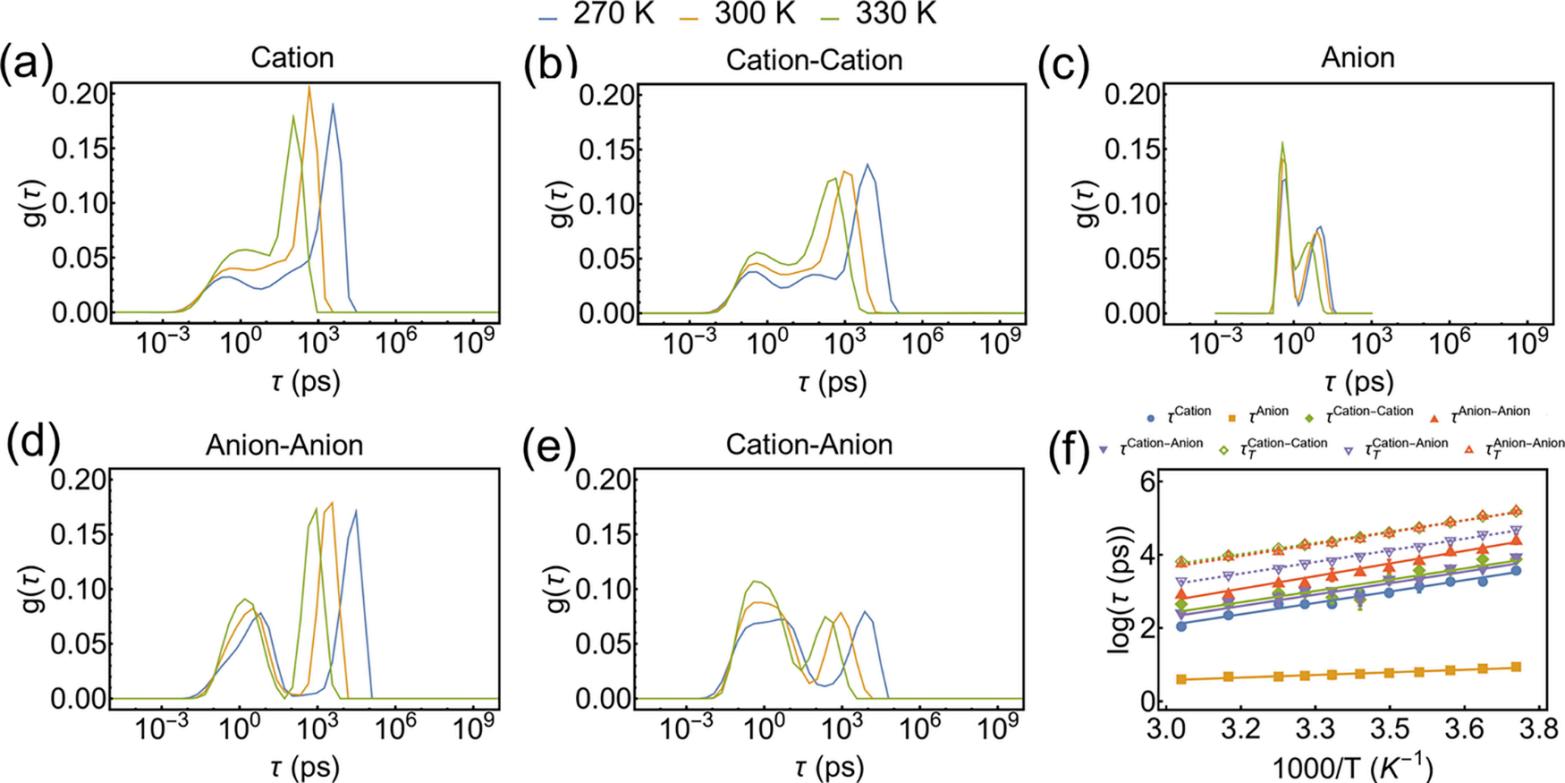
RILT-derived correlation time distributions *g*(τ) of the autocorrelation functions describing DD coupling interactions characteristic to (a) intramolecular ^1^H–^13^C for the C_2_ nucleus, (b) intermolecular ^1^H–^13^C for the C_2_ nucleus, (c) intramolecular ^19^F–^11^B, (d) intermolecular ^19^F–^19^F and (e) intermolecular ^19^F–^1^H. (f) Arrhenius plots of the slow correlation time components together with the corresponding translational correlation times derived from the Hwang–Freed model as τ_T_ = *d* ^2^/*D*_12_, where *d* is the distance of closest approach between two spins (first peak in radial distribution functions) and *D*_12_ is the sum of the self-diffusion rates. Linear fits shown as solid lines (τ) and dashed line (τ_T_).

In general, the peaks in the fast-motion region show little temperature dependence, whereas the slow correlation-time component varies strongly with temperature, consistent with previous studies.^40,41^ Notably, although the correlation time of the internal-motion peak changes only slightly with temperature, its spectral weight progressively increases with increasing temperature. This indicates that the time scale of the internal motion remains nearly unchanged, while its increasing weight enhances dipolar averaging and consequently reduces the contribution of the overall tumbling motion to the relaxation process. The Arrhenius analysis (Figure 4(f)) further reveals the dynamical processes in the system. The overall rotational motion of [BF_4_]^−^ occurs on a time scale of approximately 10 ps, substantially faster than that of the [bmim]^+^ cation, whose overall tumbling occurs on the hundreds-of-picoseconds to nanosecond time scale. All translational motions occur on longer time scales than the overall tumbling of both cation and anion. Interestingly, the activation energies associated with the tumbling motion of the [bmim]^+^ cation and the relative motions between ion pairs (cation–cation, cation–anion, and anion–anion) are very similar (see Table 1). This suggests that these processes are governed by the same underlying diffusive dynamics. Consistently, the activation energy obtained from the translational correlation time τ_*T*_ (derived from the Hwang-Freed model as τ_*T*_ = *d* ^2^/*D*_12_) shows close agreement with these values, further supporting the collective nature of these motions. In contrast, the tumbling motion of the [BF_4_]^−^ anion exhibits a lower activation energy, which can be attributed to its smaller molecular size and high tetrahedral symmetry, resulting in a lower rotational barrier. Despite this, the observation of multimodal dynamics reflects constrained motions imposed by the heterogeneous environment. Furthermore, the overall rotational motion associated with the ring carbons and the carbons proximal to the ring is slower than that of the terminal alkyl-chain carbons (see Figure S15). This trend indicates enhanced flexibility for carbon atoms located farther from the imidazolium ring.

**Table 1:** Arrhenius Fitting Parameters of Slow Correlation Time^a^

Correlation time	Slope	*E*_a_ (kJ/mol)	τ_0_ (s)
τ^Cation^	2.067 ± 0.122	39.57 ± 2.34	(7.65 ± 7.19) × 10^–17^
τ^Anion^	0.305 ± 0.046	5.83 ± 0.88	(5.11 ± 1.74) × 10^–13^
τ^Cation–Cation^	2.018 ± 0.101	38.63 ± 1.93	(2.83 ± 2.27) × 10^–16^
τ^Anion–Anion^	2.197 ± 0.104	42.07 ± 2.00	(1.43 ± 1.18) × 10^–16^
τ^Cation–Anion^	2.142 ± 0.077	41.02 ± 1.48	(7.87 ± 4.79) × 10^–17^
τ_T_^Cation–Cation^	2.016 ± 0.041	38.60 ± 0.79	(4.66 ± 1.52) × 10^–15^
τ_T_^Anion–Anion^	2.012 ± 0.075	38.53 ± 1.44	(4.21 ± 2.45) × 10^–15^
τ_T_^Cation–Anion^	2.066 ± 0.046	39.57 ± 0.89	(9.68 ± 3.46) × 10^–16^

a Slopes are obtained from linear fits of log_10_(τ) versus 1000/*T*. Activation energies *E*_a_ and pre-exponential factors τ_0_ are reported as below.

In conclusion, we report on a bottom-up computational framework for predicting relaxation rates from MD simulations tailored to investigate the complex dynamics in an IL system. Using the RILT approach, we demonstrated that NMR relaxometry probes only narrow regimes of molecular dynamics in ionic liquids, imposed by the measurement field, highlighting the limitations of simple analytical models for fitting experimental data and extracting dynamical information. We also discuss the specific contributions of intra- vs intermolecular dipolar couplings and chemical shift anisotropy to relaxation. Most correlation functions appeared to exhibit an approximately biexponential behavior. What is even more intriguing is that this is also true for intramolecular effects only. The most interesting case is the one of [BF_4_]^−^, which also shows this bimodal distribution. We attribute these findings to heterogeneous local motional restrictions imposed by the surrounding ionic environment, including steric constraints from bulky neighbors, interactions with different regions of the [bmim]^+^ cation, and variations in local ionic packing. We further highlight that the BPP-based approach, which is often used for analysis,^29,39^ grossly misrepresents the underlying dynamics even if just an average correlation time is assumed. Lastly, we envision that accurate predictions of nuclear polarization lifetimes in solution can become a vehicle for benchmarking force-fields,^44–46^ complementing classical validation approaches based on static properties, such as density and radial distribution functions, and dynamic quantities, such as viscosity, diffusion, and dielectric response. Quantitative match between MD-predicted and experimental NMR relaxation rates over multiple nuclear species and experimental conditions will confirm the accuracy of a tested force field and, thus, will provide access to a significantly broader window of structural and dynamical features than the ones inferred using the BPP model.

## Materials and Methods

### Sample Preparation and NMR Measurements

Neat [bmim]­[BF_4_], purchased from Acros Organics, was transferred into the outer compartment of a 5 mm coaxial NMR tube under an inert argon atmosphere to avoid degradation. A 2 mm inner insert containing D_2_O was used exclusively for magnetic field locking and shimming. The inversion–recovery pulse sequence was employed to measure the spin–lattice relaxation times (*T*_1_) of ^13^C and ^19^F.

### Simulations

Molecular dynamics simulations were performed using GROMACS 2025.3 with a charge-scaled OPLS force field.^47,48^ A cubic box with a side length of 53.7 Å was constructed, containing 500 [bmim]^+^ cations and 500 [BF_4_]^−^ anions, corresponding to a target density of 1.21 g/mL. Energy minimization was carried out using the steepest descent algorithm for 5000 steps. The system was equilibrated for 5 ns under NPT conditions using the V-rescale thermostat and the Berendsen barostat at a constant pressure of 1.0 bar, with temperatures ranging from 270 to 330 K. Production simulations were then performed under the same NPT conditions with a time step of 1 fs.^14^ The production trajectory length ranged from 20 to 120 ns depending on temperature, and was chosen to be longer than five times the estimated correlation time. For the main autocorrelation-function and RILT analyses, trajectories were saved every 0.1 ps. For the [BF_4_]^−^ reorientational dynamics, additional shorter simulations with a saving frequency of 1 fs were performed to better resolve the rapid rotational motion. Five independent MD simulations with different initial configurations were carried out, and the results were averaged over the five trajectories. The error bars reported for the simulation results are derived from the variation among these five independent trajectories. Chemical shift tensors were computed with Gaussian employing the B3LYP functional and the cc-pVTZ basis set.^49,50^ A detailed description of the chemical shift tensor calculations is provided in the Supporting Information.

## Supplementary Material



## Notes

Additional details describing our computational workflow, CSA relaxation rate predictions and results for all carbon and fluorine nuclei (PDF)
