# Supplementary material for: Multimodal Dynamics in Ionic Liquids Revealed by Molecular-Dynamics-Guided Multinuclear NMR Relaxation Analysis
Source: J Phys Chem Lett. 2026 Jul 13;17(29):8342–9. doi: 10.1021/acs.jpclett.6c02150 (PMC13403297; doi:10.1021/acs.jpclett.6c02150)
Supplement: Supplementary file 1 [file jz6c02150_si_001.pdf]

Supporting Information:

Multimodal Dynamics in Ionic Liquids Revealed by Molecular-Dynamics-Guided Multinuclear NMR Relaxation Analysis

Yanan Li[†], Florin Teleanu[*,†,‡], Federico Civaia[¶], Christoph Scheurer[¶] and Alexej Jerschow[*,†]

[†]*Department of Chemistry, New York University, New York, New York 10003, United States*

[‡]*ELI-NP, "Horia Hulubei" National Institute for Physics and Nuclear Engineering, 30 Reactorului Street, Bucharest-Magurele 077125, Ilfov, Romania*

[¶]*Fritz Haber Institute of the Max Planck Society, Berlin 14195, Germany*

E-mail: alexej.jerschow@nyu.edu; ft2287@nyu.edu

Contents of the Supporting Information

This Supporting Information contains:

- results showcasing the deviations of DD interactions from the BPP and Hwang Freed model
- details of the relaxation rate calculations due to CSA interaction
- details of the regularized inverse Laplace transform procedure
- comparison between MD-derived and experimental diffusion rates
- predicted contributions of relaxation interactions for all carbon nuclei of [bmim] at different temperatures
- predicted contributions of relaxation interactions for all fluorine nuclei of $[BF_4]^-$ at different temperatures
- RILT results for all carbon and fluorine nuclei at different temperatures
- Arrhenius plot of the slow tumbling component of the intramolecular DD interaction for all carbon nuclei of [bmim]

1 Deviations from the BPP model and the Hwang-Freed model

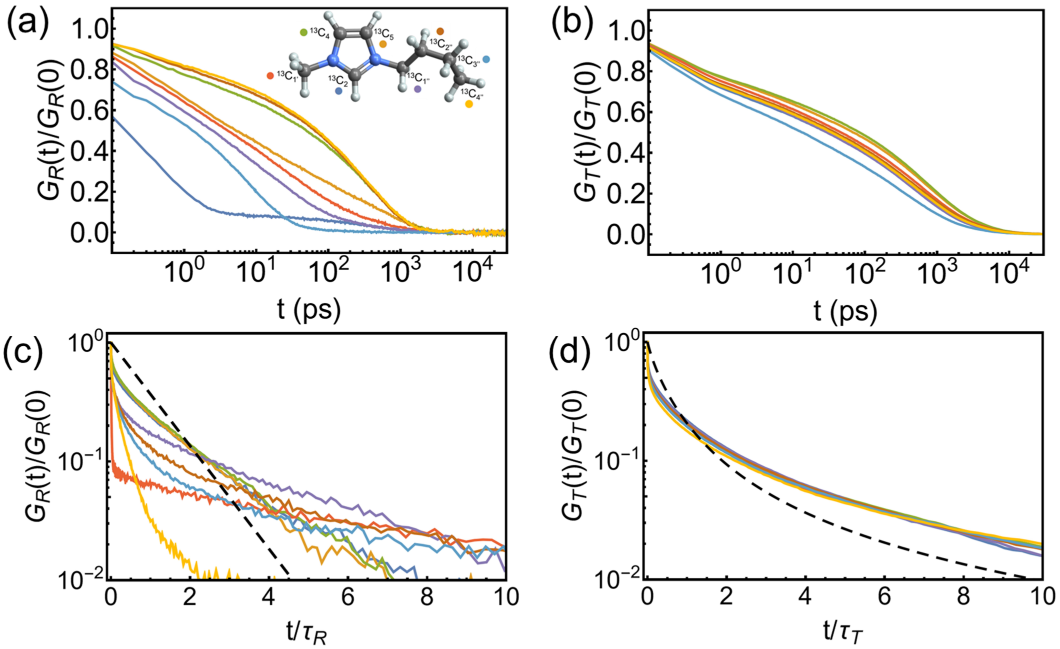

Figure S1: (a) Normalized autocorrelation functions of intramolecular ^{13}C-^{1}H dipole-dipole interactions as a function of time for all ^{13}C sites at 300 K. (b) Normalized autocorrelation functions of intermolecular ^{13}C-^{1}H dipole-dipole interactions as a function of time for all ^{13}C sites at 300 K. (c) Intramolecular ^{13}C-^{1}H autocorrelation functions as a function of t/τ_R; the dashed line represents the BPP model. (d) Intermolecular ^{13}C-^{1}H autocorrelation functions as a function of t/τ_T; the dashed line corresponds to the Hwang–Freed model. The color coding corresponds to the carbon-site labeling shown in panel (a).

2 Calculation of CSA tensors

As classical MD simulations do not explicitly resolve the electronic structure, a simplified strategy was adopted to estimate the chemical shift anisotropy (CSA) contribution to relaxation. Two approximations were introduced. First, instead of evaluating the CSA tensor at every MD frame, 100 representative configurations were extracted from the equilibrated trajectories to account for configurational fluctuations. Second, the rotational correlation time of the CSA tensor was approximated using the rotational dynamics of specific bond vectors.

For the selected configurations, the nuclear shielding tensors were calculated at the B3LYP/cc-pVTZ level using Gaussian. Each shielding tensor $\boldsymbol{\sigma}$ was decomposed into its symmetric and antisymmetric components:

$$\boldsymbol{\sigma}_{\text{sym}} = \frac{1}{2}\left(\boldsymbol{\sigma} + \boldsymbol{\sigma}^{\text{T}}\right) - \frac{1}{3}\text{Tr}(\boldsymbol{\sigma})\mathbf{I}. \tag{S1}$$

$$\boldsymbol{\sigma}_{\text{anti}} = \frac{1}{2}\left(\boldsymbol{\sigma} - \boldsymbol{\sigma}^{\text{T}}\right). \tag{S2}$$

The Frobenius norms of the symmetric and antisymmetric components were evaluated as

$$\|\Delta\sigma_{\text{sym}}\|_F = \sqrt{\sum_{i,j}\left(\Delta\sigma_{\text{sym},ij}\right)^2}, \tag{S3}$$

$$\|\Delta\sigma_{\text{anti}}\|_F = \sqrt{\sum_{i,j} (\Delta\sigma_{\text{anti},ij})^2}. \tag{S4}$$

As illustrated in Fig. S2, the principal axes of the CSA tensors are consistently aligned along specific chemical bonds (e.g., C-N, C-C, C-H, or B-F). This observation suggests that the orientational modulation of the CSA tensor is effectively governed by the rotational motion of the corresponding bond vector. Accordingly, the bond-vector rotational correlation time was used as a proxy for the CSA tensor reorientation time. The corresponding reorientational autocorrelation function was calculated as

$$G_{\text{CSA}}(t) = \langle P_2[\hat{\mathbf{u}}(t_0) \cdot \hat{\mathbf{u}}(t_0 + t)] \rangle_{\text{origins, residues}}, \tag{S5}$$

where $\hat{\mathbf{u}}(t)$ is the normalized bond vector used to approximate the principal reorientation axis of the CSA tensor, and $P_2(x) = (3x^2 - 1)/2$ is the second-order Legendre polynomial.

Given that the CSA contribution to the ^{13}C and ^{19}F relaxation rates in [bmim][BF_4] is relatively small compared to dipole-dipole interactions, this simplification is considered reasonable for the current system. However, for systems in which CSA dominates the relaxation mechanism, such an approximation should be applied with caution. In principle, a more rigorous treatment would involve computing the CSA tensor for each MD snapshot, for example via machine-learning-accelerated electronic structure methods, and directly evaluating its rotational autocorrelation function. Such an approach would provide a more complete description of CSA-driven relaxation. Nevertheless, in [bmim][BF_4], CSA does not constitute the primary relaxation pathway, and the present system is not ideally suited for developing or validating a machine-learning model specifically targeting CSA tensor prediction.

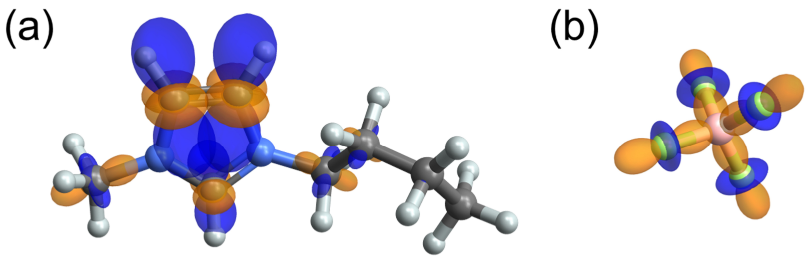

Figure S2: (a) Representation of the symmetric component of the CSA tensors for ^{13}C nuclei in [bmim]$^+$ and (b) for ^{19}F nuclei in [BF_4]$^-$.

Fig. S3-S5 show the Frobenius norms of the symmetric and antisymmetric components of the CSA tensors for ^{13}C nuclei in [bmim]$^+$ and ^{19}F nuclei in [BF_4]$^-$. The dashed lines indicate the averaged values, which were used as prefactors in the relaxation-rate expressions.

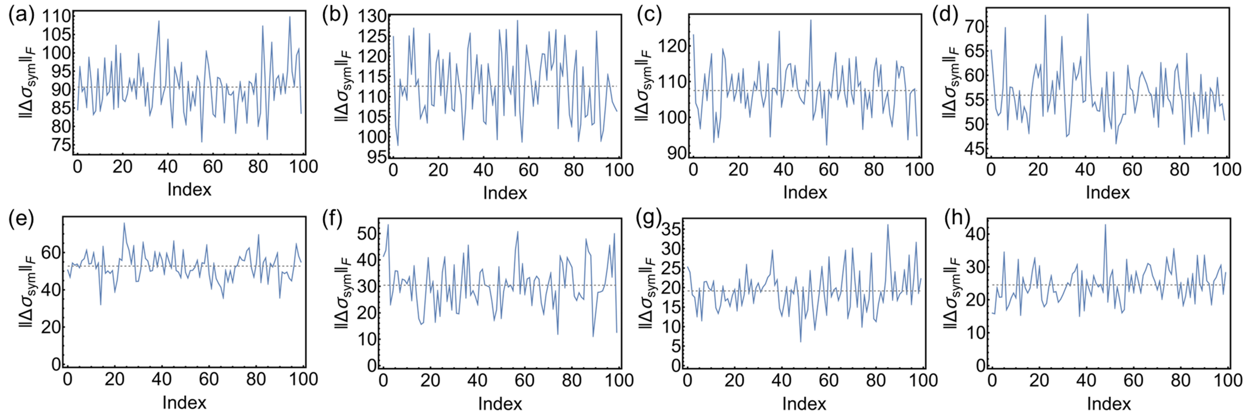

Figure S3: Frobenius norms of the symmetric components of the CSA tensors for (a) $^{13}C_2$, (b) $^{13}C_4$, (c) $^{13}C_5$, (d) $^{13}C_{1'}$, (e) $^{13}C_{1''}$, (f) $^{13}C_{2''}$, (g) $^{13}C_{3''}$, and (h) $^{13}C_{4''}$ in $[\mathrm{bmim}]^+$.

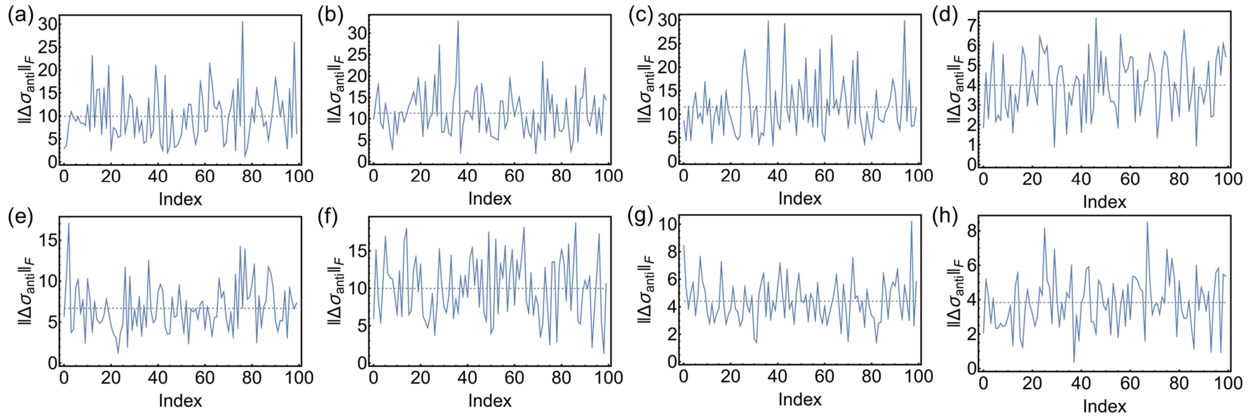

Figure S4: Frobenius norms of the antisymmetric components of the CSA tensors for (a) $^{13}C_2$, (b) $^{13}C_4$, (c) $^{13}C_5$, (d) $^{13}C_{1'}$, (e) $^{13}C_{1''}$, (f) $^{13}C_{2''}$, (g) $^{13}C_{3''}$, and (h) $^{13}C_{4''}$ in $[\mathrm{bmim}]^+$.

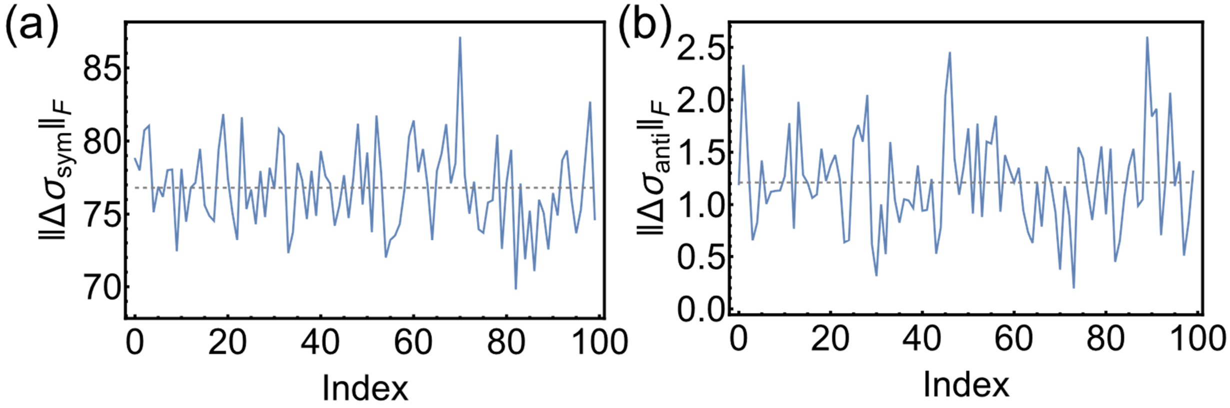

Figure S5: Frobenius norms of the (a) symmetric and (b) antisymmetric components of the CSA tensors for ^{19}F in $[BF_4]^-$.

3 Calculation of regularized inverse Laplace transform

The `iltpy` Python package was used to perform the regularized inverse Laplace transform (RILT) calculations [1]. All parameters were set to their default or recommended values [2], except for the regularization parameter α_{00}. As the primary regularization parameter, α_{00} plays a critical role in controlling the strength of regularization and therefore directly influences the balance between data reliability and solution smoothness. To determine an appropriate value of α_{00}, the S-curve method was employed (Fig. S6) [3, 4]. In this approach, the residual norm is analyzed to identify a stable operating regime. Based on this analysis, $\alpha_{00} = 10^{-4}$ was selected, corresponding to a slope close to 0.1 in the S-curve. This choice provides a balanced solution that avoids both noise amplification and excessive smoothing of the correlation-time distribution. To further illustrate the effect of the regularization parameter, Fig. S7 compares the extracted correlation-time distributions, $g(\tau)$, obtained using different values of α_{00}. At $\alpha_{00} = 10^{-4}$, the RILT spectrum exhibits clear bimodal dynamic components. When α_{00} is increased to 10^{-1}, these components are over-smoothed into a single broad peak, accompanied by a much larger residual norm, indicating over-regularization. Under even stronger regularization, such as $\alpha_{00} = 10^5$, the distribution becomes excessively broadened and the residual norm increases dramatically, reaching approximately 10. In contrast, when α_{00} is decreased to 10^{-7}, additional narrowly separated peaks appear, which are likely associated with noise amplification and under-regularization. These comparisons support the selection of $\alpha_{00} = 10^{-4}$ and indicate that the bimodal features are robustly supported by the autocorrelation data rather than being artifacts of either over- or under-regularization.

The trajectory saving interval and total simulation length define the approximate lower and upper bounds of the artifact-free window in the RILT analysis, respectively, which we estimate as $10t_{\mathrm{timestep}}$ and $t_{\mathrm{MD}}/5$ as a rather conservative range. In the present work, the slow dynamic components identified in the RILT spectra fall entirely within this artifact-free window. The interpretation of the fast components, however, requires additional caution. For these fast components, the peak centers and a substantial portion of the peak areas remain within the artifact-free window, while part of the peak widths extends beyond this range. Therefore, the apparent broadening of the fast-component peaks is likely influenced, at least in part, by residual early-time artifacts. Given the computational cost and memory limitations associated with simultaneously achieving long trajectories and high-frequency trajectory saving, the present simulations represent the highest practical temporal resolution and trajectory length achievable under our current computational conditions.

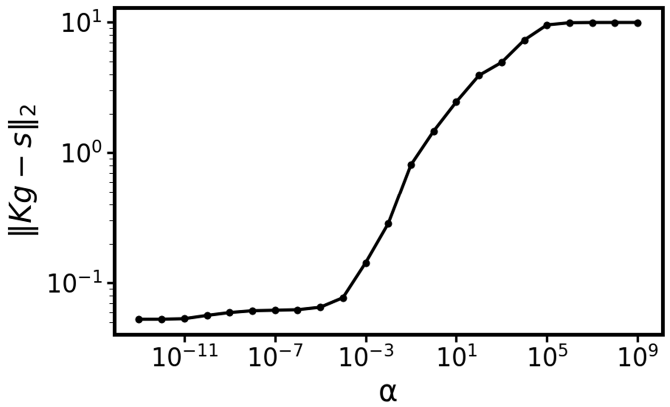

Figure S6: Log-log plot of the fitting-error norm as a function of the regularization parameter.

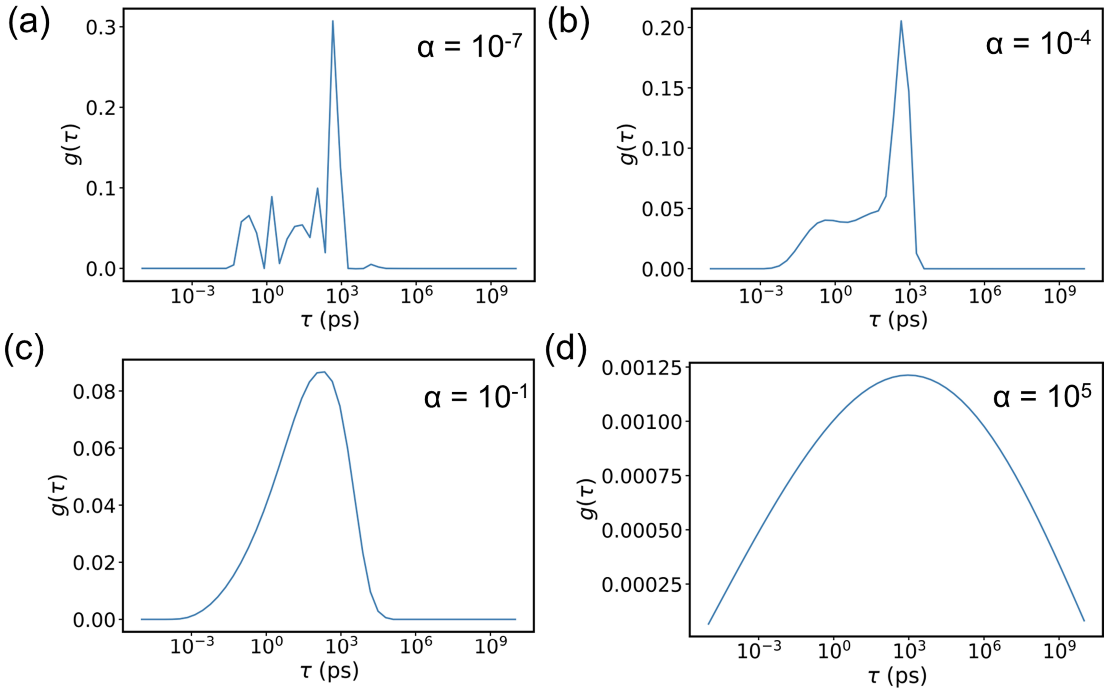

Figure S7: Correlation-time distributions, $g(\tau)$, obtained from the regularized inverse Laplace transform using different regularization parameters: (a) $\alpha = 10^{-7}$, (b) $\alpha = 10^{-4}$, (c) $\alpha = 10^{-1}$, and (d) $\alpha = 10^5$.

4 Diffusion rate predictions

To assess the reliability of the OPLS force field[5] in describing the dynamical properties, the mean-squared displacements (MSDs) of both the cations and anions were calculated and compared with previously reported experimental diffusion data (see Fig. S8) [6]. Over the investigated temperature range, excellent agreement was observed, confirming that the employed force field provides an accurate description of the inoic liquids dynamics.

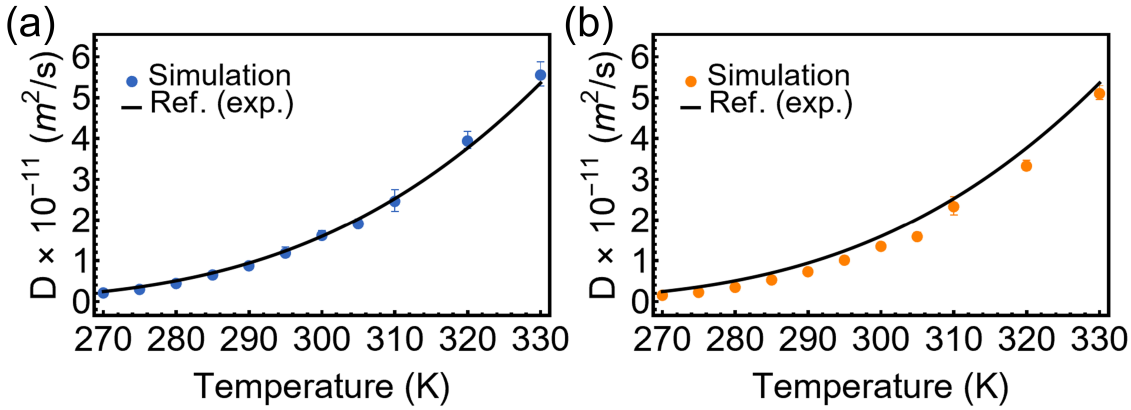

Figure S8: Self-diffusion coefficients of (a) the $[\text{bmim}]^+$ cation and (b) the $[\text{BF}_4]^-$ anion in $[\text{bmim}][\text{BF}_4]$. Symbols represent values obtained from the present MD simulations, while the solid lines correspond to interpolated functions of the experimental pulse-field-gradient spin-echo data [6].

5 Fitting of the temperature-dependent ^{13}C relaxation rates

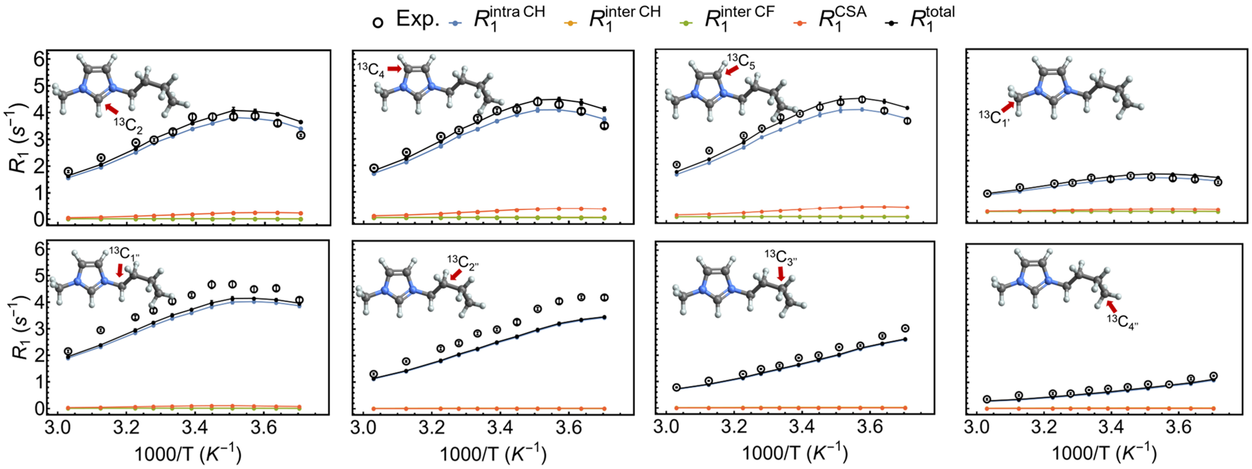

Figure S9: Temperature dependence of the identified relaxation contributions (lines), whose sum reproduces the overall ^{13}C relaxation profile in $[\text{bmim}][\text{BF}_4]$.

6 Fitting of the temperature-dependent ^{19}F relaxation rates

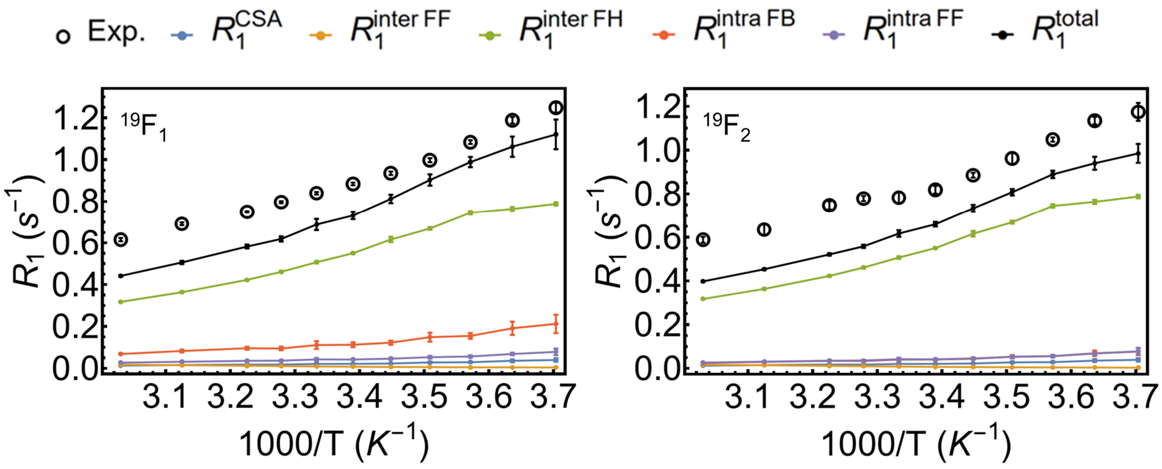

Figure S10: Temperature dependence of the identified relaxation contributions (lines), whose sum reproduces the overall ^{19}F relaxation profile in [bmim][BF_4].

7 Regularized inverse Laplace transform spectra

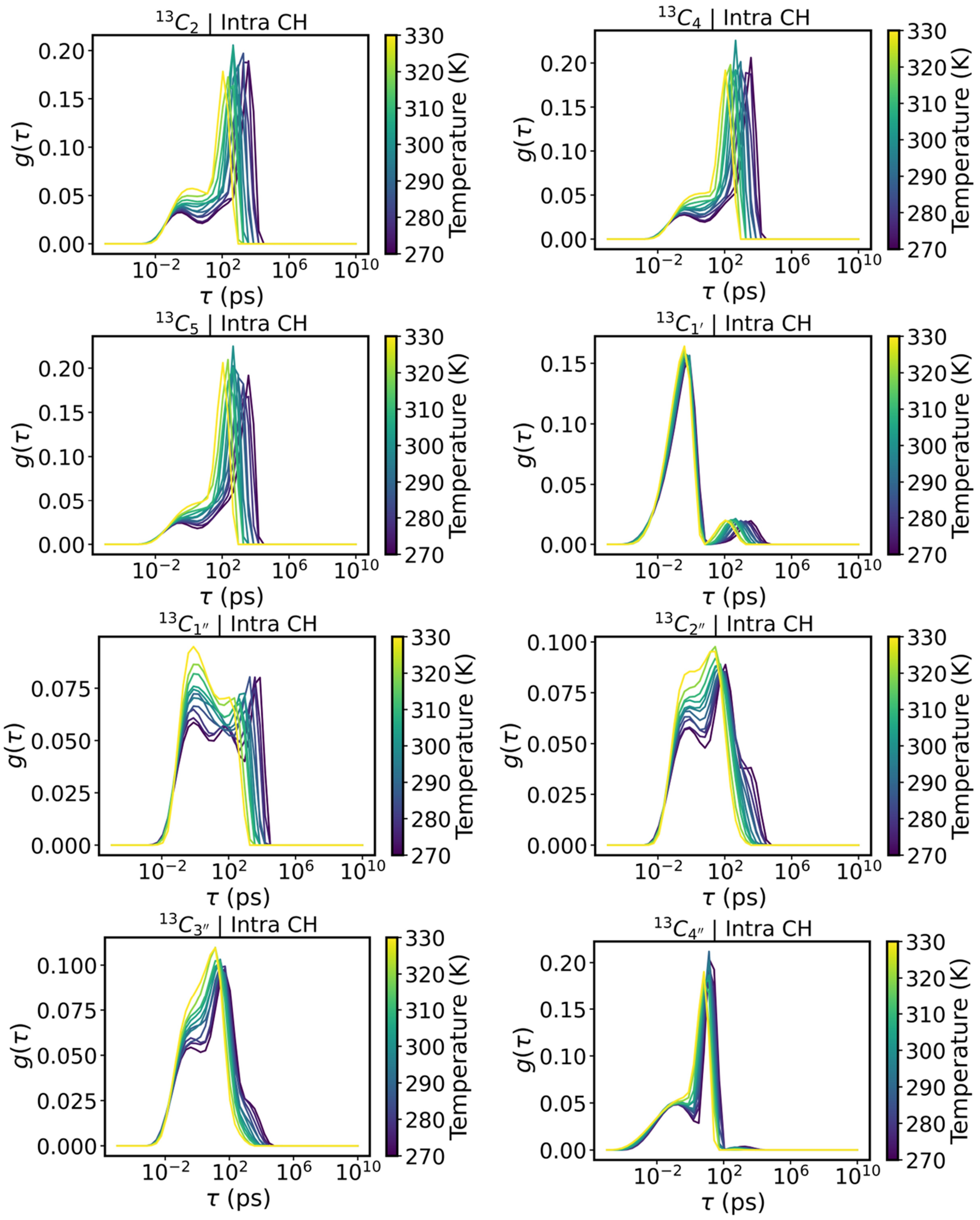

Figure S11: RILT spectra of the autocorrelation functions associated with intramolecular C-H dipole-dipole interactions of ^{13}C at different temperatures.

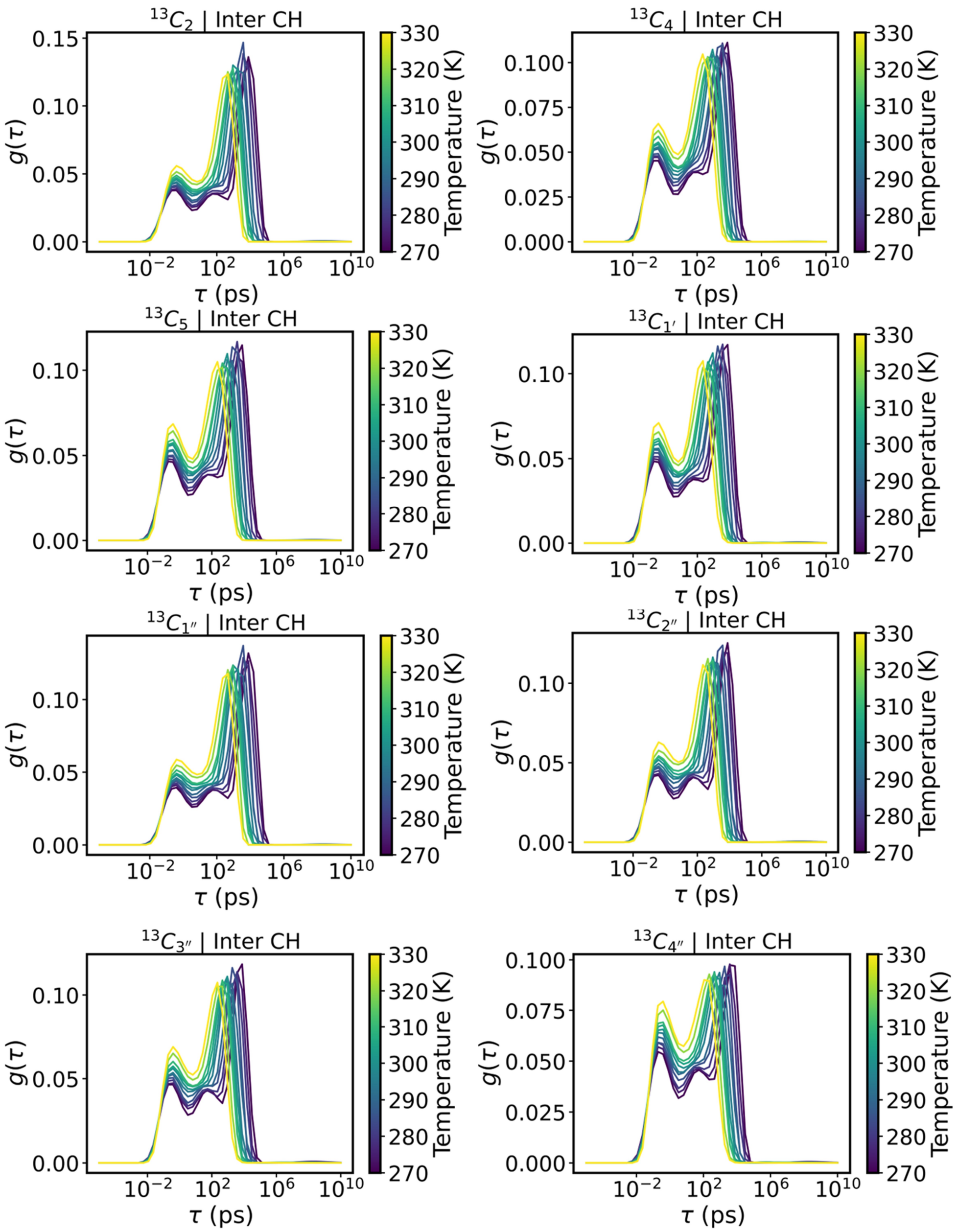

Figure S12: RILT spectra of the autocorrelation functions associated with intermolecular C-H dipole-dipole interactions of ^{13}C at different temperatures.

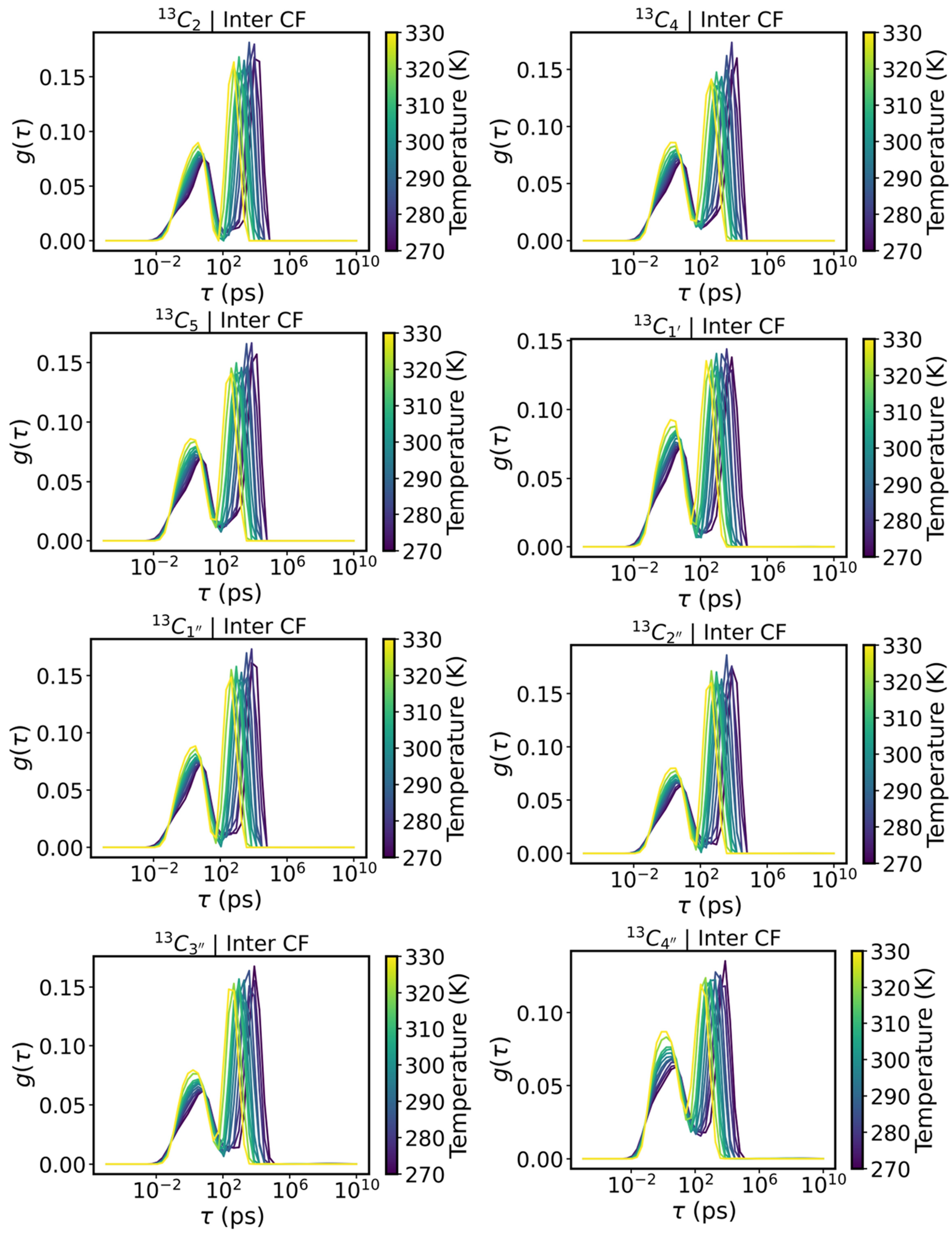

Figure S13: RILT spectra of the autocorrelation functions associated with intermolecular C-F dipole-dipole interactions of ^{13}C at different temperatures.

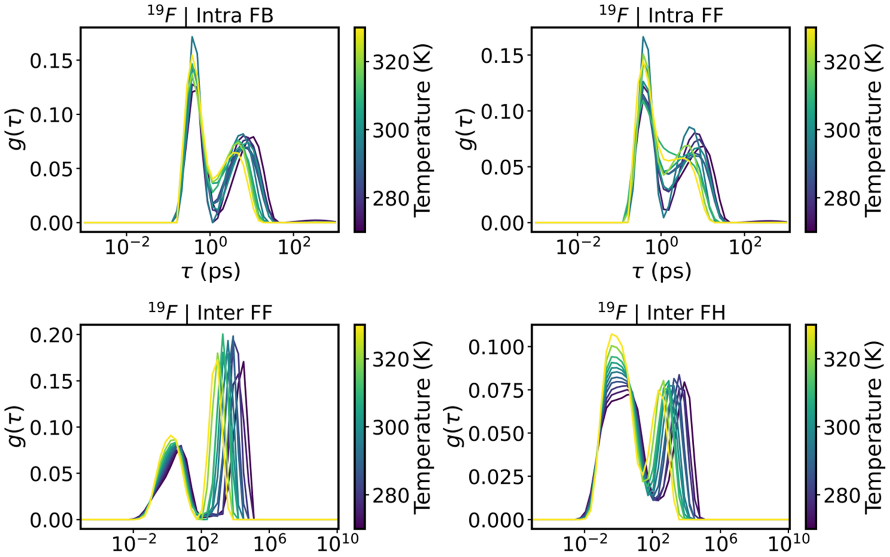

Figure S14: RILT spectra of the autocorrelation functions associated with dipole-dipole interactions of ^{19}F at different temperatures.

8 Arrhenius plot

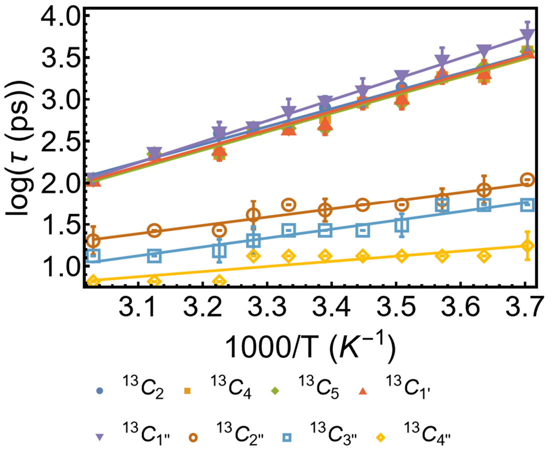

Figure S15: Arrhenius plots of the slow correlation time component of the intramolecular ^{13}C-^{1}H dipole interactions for all ^{13}C sites, with linear fits.

References

(1) Daniel, D. T.; Bartsch, C.; Bereck, F. P.; Köcher, S.; Scheurer, C.; Granwehr, J. ILTpy, version 1.1.0, 2025.

(2) Granwehr, J.; Roberts, P. J. Inverse Laplace transform of multidimensional relaxation data without non-negativity constraint. *J. Chem. Theory Comput.* **2012**, *8*, 3473–3482.

(3) Fordham, E.; Sezginer, A.; Hall, L. Imaging multiexponential relaxation in the (y, LogeT1) plane, with application to clay filtration in rock cores. *J. Magn. Reson. Ser. A* **1995**, *113*, 139–150.

(4) Granwehr, J.; Roberts, P. J. Inverse Laplace transform of multidimensional relaxation data without non-negativity constraint. *J. Chem. Theory Comput.* **2012**, *8*, 3473–3482.

(5) Doherty, B.; Zhong, X.; Gathiaka, S.; Li, B.; Acevedo, O. Revisiting OPLS force field parameters for ionic liquid simulations. *J. Chem. Theory Comput.* **2017**, *13*, 6131–6145.

(6) Tokuda, H.; Hayamizu, K.; Ishii, K.; Susan, M. A. B. H.; Watanabe, M. Physicochemical properties and structures of room temperature ionic liquids. 1. Variation of anionic species. *J. Phys. Chem. B* **2004**, *108*, 16593–16600.
